# Spectrum of *TP53* Mutations in BRCA1/2 Associated High-Grade Serous Ovarian Cancer

**DOI:** 10.3389/fonc.2020.01103

**Published:** 2020-07-16

**Authors:** Ulyana A. Boyarskikh, L. F. Gulyaeva, A. M. Avdalyan, A. A. Kechin, E. A. Khrapov, D. G. Lazareva, N. E. Kushlinskii, A. Melkonyan, A. Arakelyan, Maxim Leonidovich Filipenko

**Affiliations:** ^1^Institute of Chemical Biology and Fundamental Medicine, Siberian Branch of the Russian Academy of Sciences (SB RAS), Novosibirsk, Russia; ^2^Institute for Medicine and Psychology, Novosibirsk State University, Novosibirsk, Russia; ^3^Institute of Molecular Biology and Biophysics, Federal Research Center of Fundamental and Translational Medicine, Novosibirsk, Russia; ^4^City Clinical Hospital No. 40, Moscow, Russia; ^5^Altai Territorial Cancer Control Center, Barnaul, Russia; ^6^N.N. Blokhin National Medical Research Center of Oncology, Moscow, Russia; ^7^Group of Bioinformatics, Institute of Molecular Biology, Armenian National Academy of Sciences (NAS RA), Yerevan, Armenia

**Keywords:** TP53 somatic mutations, p53 expression, gain of function, loss of function, *BRCA1/2 carriers*, ovarian cancer

## Abstract

**Objective:** Mutations in TP53 lead to loss of function (LOF) or gain of function (GOF) of the corresponding protein p53 and produce a different effect on the tumor. Our goal was to determine the spectrum of somatic *TP53* variants in *BRCA1/2* associated high-grade serous ovarian cancer (HGSOC).

**Methods:** The population under study comprised of HGSOCs with pathogenic variants in *BRCA1* (*n* = 78) or *BRCA2* (*n* = 21). Only chemo-naive and platinum-sensitive patients were included in this study. The case group of the IARC database (*n* = 1249) with HGSOC not stratified by BRCA status was used as a reference. A custom NGS panel was used for sequencing *TP53* and mutational hot-spots of other genes, and p53 expression was evaluated by immunohistochemistry for 68 cases of HGSOCs.

**Results:** Somatic *TP53* variants (95) or inhibition of wild-type p53 expression (3) were observed in 98 cases. The sample with normal p53 had *CDKNA1* variants. The frequency of truncating variants was significantly higher than in the reference cohort (30.3 vs. 21.0%, *p* = 0.01). Most of the samples (41/68) demonstrated low (or absent) expression of p53, and 17 samples overexpressed p53. LOH was typical for TP53 nonsense variants (14/15). In total, 68/95 samples were LOH positive and showed LOH in all tumorous cells, thus indicating the driver effect of *TP53* mutations. Three specimens had *KRAS, BAX, APC*, and *CTNNB1* subclones variants.

**Conclusion:** High frequency of *TP53* truncating variants, the low expression of mutant p53, and low incidence of oncogene mutations show potential GOF properties of p53 to be poorly represented in BRCA1/2 associated HGSOC.

## Introduction

TP53 is the most frequently mutated gene in human cancer (1). About 75% of TP53 variants are missense substitutions (2). Other alterations include frameshift indels and nonsense variants (20%) and infrequent substitutions in splicing sites, inframe indels, and silence variants. Frameshift indels and nonsense variants always lead to the null p53 phenotype. In contrast, missense variants result in a full-size mutant p53, which can stably express and have a different effect on the tumor. The loss or decrease in p53 transcriptional activity (loss of function, LOF) is a common property of all p53 mutants associated with cancer. Along with LOF, a number of additional p53 action scenarios are possible. Specifically, variants of “separation of functions” are described (3). In this case, the mutant retains some pro-survival functions and selectively loses tumor-suppressive activity of p53 wild-type, as shown for the apoptosis-deficient R175P mutant p53 (4). Finally, some mutants acquire new oncogenic properties (gain of function, GOF). Studies of cancer cell lines and animal cancer models have shown that GOF TP53 variants can contribute to chemotherapy resistance and cancer progression (3, 5–8). Thus, mutant p53 plays a complex role in tumorigenesis that varies depending on both the mutation type and tumor origin.

The prevalence of missense variants and the fact that GOF mutants of TP53 promote tumor progression led to speculation about the positive selection of GOF variants during carcinogenesis.

High-grade serous ovarian carcinoma (HGSOC) is the most common type of ovarian cancer characterized by difficulties in early detection and high mortality rates. HGSOC is a unique type of cancer in terms of the prevalence of TP53 mutations. Almost all HGSOC tumors (95%) carry somatic TP53 variants. A comparable incidence of TP53 mutations is observed only in serous endometrial carcinomas (89%) and basal subtype breast tumors (88%) (1, 9). Almost 20% of HGSOCs are associated with germline BRCA1/2 variants (10). BRCA proteins is involved in the repair of DNA double-strand breaks by homologous recombination (HR) (11). The complete loss of BRCA1/2 function leads to disruption of the HR-based DNA repair and, as a result, to the large-scale genomic instability (12). In this case, alterations in TP53 (or other cell cycle control genes) are mandatory for the cell viability. Otherwise, genomic instability results in cell-cycle arrest and apoptosis. For HGSOC with an HR deficiency, we assumed LOF of p53 to be selected more frequently than GOF. To test this hypothesis, we determined the frequencies of various types of TP53 variants in the cohort of BRCA1/2-deficient HGSOCs. To evaluate the involvement of other genes in BRCA1/2-associated HGSOC pathogenesis, we tested these tumors for the frequent somatic variants in oncogenes.

## Materials and Methods

### Patients and Samples

Samples were obtained from patients with relapse of high-grade serous ovarian adenocarcinomas after first-line treatment, undergoing tumor testing under the program “Improving the system of molecular genetic diagnosis of cancer in the Russian Federation” (http://www.cancergenome.ru). Ethical approval for this project was obtained from the Institute of Molecular Biology and Biophysics Ethics Committee (Protocol: # 1 dated March 14, 2017). Written informed consent was obtained from all subjects.

All patients underwent primary cytoreductive surgery, followed by adjuvant platinum-based chemotherapy as first-line treatment. Eligibility criteria for the study were (1) histologically verified diagnosis of high-grade serous ovarian (or fallopian tube) adenocarcinomas; (2) lack of neo-adjuvant therapy; (3) platinum sensitivity (the time from adjuvant platinum-based treatment to cancer relapse (platinum-free interval, PFI) was > 6 months), (4) available blood sample and FFPE tissue from the primary tumor; and (5) germline pathogenic variants in *BRCA1* or *BRCA2*. The FFPE primary tumor blocks were sectioned and stained with hematoxylin and eosin (H&E). Tumor regions on H&E stained slides were marked, and the percentage of tumor cells was estimated. Samples containing at least 10% of tumor cells were selected for this study. DNA was extracted from marked regions separated by manual macrodissection from three unstained 10 μm-thick FFPE sections. DNA isolation was carried out using alkaline lysis followed by DNA extraction from the precipitate, as described previously (13). DNA from blood leukocytes was extracted using an in-house method involving cell lysis using 10% SDS-containing buffer, proteinase K treatment, protein extraction using phenol-chloroform, and isopropanol precipitation of the DNA. All DNA samples were screened for sufficient quantity using the PCR based QC Kit (Kapa Biosystems). Germline *BRCA1/2* variants were determined by NGS of gene coding sequences and splicing site regions, as described previously (14). DNA samples from both leukocyte and FFPE tumors were sequenced. As a result, 99 tumors were revealed, with 78 being *BRCA1* germline mutation carriers and 21 – *BRCA2* (the full list of the BRCA variants is given in the Supplementary Table 1).

### NGS Panel and Data Analysis

DNA target sequencing was performed using the PCR-based custom NGS panel called CCMSeq (Common Cancer Mutations). CCMSeq panel was designed for analyzing multiple genome regions that are commonly mutated in a variety of cancer types. This panel covers 8.6 kilobases across all 11 exons and adjacent intron regions of *TP53*, as well as coding regions of cancer-related genes that carry the most frequent somatic variants. These regions were selected based on the whole genome (or exome) sequencing data cataloged in the COSMIC database for colon, stomach, lung, breast, and ovary cancers. Regions carrying somatic mutations with a prevalence of at least 2% in two or more cancer types were included in the CCMSeq panel. In total, loci of 50 genes were selected for the CCMSeq panel (Supplementary Table 2). These selected targets were amplified using two multiplex PCRs with the amplicon library preparation procedure described previously (14). Normalized amplicon libraries were sequenced on a MiniSeq platform (Illumina) using a MiniSeq High Output Reagent Kit (300 cycles).

The procedure of NGS data analysis was similar to those described previously (14) with some modification: short-variant calling was performed by Pisces (https://github.com/Illumina/Pisces, somatic mode). To filter the false positive variants, those with less than six reads of alternative alleles were discarded. The coverage (median coverage of one library amplicon) ranged from 163 to 7273 reads, with median and Q1-Q3 values of 1087 and 735-2037 reads, respectively. The following variant types were taken into consideration: (1) frameshift, stop gained, stop lost, start lost, splice acceptor, splice donor variants; (2) missense and splice region variants that according to 1000Genomes project have a population frequency less than 0.5%; (3) variants listed in the COSMIC database; (4) variants registered in the ClinVar database as “Pathogenic”/”Likely pathogenic.” Differentiation between somatic and germline variants was performed by sequencing both leukocyte and tumor DNA samples. The variants with low variant allele frequency (VAF) were filtered out. The cut-off of VAF was 5% - for tumor DNA and 20% - for leukocyte DNA. DNA samples with somatic variant VAF <15% were sequenced twice.

The NGS data supporting this study have been deposited in the National Center for Biotechnology Information (NCBI)'s Sequence Read Archive (SRA, https://www.ncbi.nlm.nih.gov/sra) with BioProject ID PRJNA612603 and can be accessed at https://www.ncbi.nlm.nih.gov/sra/PRJNA612603.

### Classification of TP53 Variants

*TP53* variants were classified into three categories: GOF, LOF, and “unclassified,” according to the criteria described by Brachova et al. (15). Specifically, *TP53* variants were defined as GOFs, based on experimental studies that showed the oncogenic properties of mutant p53. Eight *TP53* mutations were considered as GOF: P151S, Y163C, R175H, L194R, Y220C, R248Q/W, R273C/H/L, R282W. Nonsense and frameshift variants leading to significant disruptions in the p53 translation were classified as LOF. The remaining missense and splice site variants, the function of which is not yet well known, were categorized as “unclassified” variants.

Additionally, we used the International Agency for Research on Cancer (IARC) *TP53* database to further characterize all missense variants by the transcriptional and GOF activity in corresponding mutants (http://p53.iarc.fr/DownloadDataset.aspx (Files: somatic Mutation Data IARC *TP53* Database, R20.txt, and functional Assessment IARC *TP53* Database, R20.txt)). Characterization of GOF missense variants was performed, as described previously (16). As a reference group of variants specific for HGSOC the variant set of the IARC *TP53* database (File: somatic Variant Data IARC *TP53* Database, R20.txt) non-stratified by *BRCA* status, was used. Only the cases with morphology corresponding to adenocarcinoma and cystadenocarcinoma (1249 in total) were selected. Ethnicity and BRCA status were not indicated for most samples. The reference set of *TP53* variants did not contain silent variants.

### Pathomorphological and Immunohistochemical Assessment

The percentage of tumor cells relative to other cells was estimated independently by two pathologists using the same slides stained with hematoxylin and eosin. In six tumors with extensive inflammatory infiltration and/or diffuse stromal invasion, epithelial cells were labeled with a pan-cytokeratin antibody cocktail (antibody clone AE1/AE3, M3515, Dako, CA, USA) for a more accurate estimation of tumor cell percentage. Finally, the percentage of tumor cells was calculated as the average value of both measurements. The mean difference between the measurements was 6 ± 4%. The percentage of tumor cells ranged from 10 to 95% with the median and Q1-Q3 values of 60% and 45–75%, respectively (the actual percentage of tumor cells is given in Supplementary Table 1). FFPE tissue sections were subject to immunohistochemistry for p53 using a commercially available mouse monoclonal anti-human p53 antibody (clone DO-7, M7001, Dako, CA, USA) at the dilution of 1:50. Staining was performed on a whole section using a Ventana BenchMark GX autostainer (Roche) according to the standard protocol in automatic mode. Stained slides were examined by an experienced surgical pathologist who was not aware of molecular data. The percentage of cells with positive nuclear staining was estimated and subdivided into three categories: ≥ 70% positively stained nuclei (High); >10% and < 70% stained nuclei (Intermediate); ≤ 10% positively stained nuclei (Low).

### Statistical Methods

Statistical analyses were performed applying R 2.13.1 statistical software. Results were compared using the χ^2^ test.

## Results

### Patient Characteristics

Ninety nine patients with HGSOC (4 with fallopian tube carcinoma and 95 with ovarian carcinoma) were enrolled in this study. In most patients (67/99, 67.0%), ovarian cancer was diagnosed at the age > 50 years. The median and Q1-Q3 values of age were 54 (49-60) years. The cohort of patients with HGSOC comprised cases with II (15), (III) 71, and IV (13) FIGO stages. Tumor grade was determined as G2 in 19 cases, G3 in 73 cases, and undetermined for others cases. All 99 patients underwent primary cytoreductive surgery, followed by first-line platinum-based chemotherapy, most with carboplatin-paclitaxel regimen (88). Other treatments included cisplatin-doxorubicin-cyclophosphamide (5), or not specified (platinum-based) (6). All patients experienced a complete or partial response after adjuvant therapy with platinum-free interval (PFI) > 6 months. For 32/99 patients, PFD was specified and ranged from 7 up to 21 months (median (Q1-Q3), 13 (11-16) months). All patients were carriers of a germline pathogenic BRCA1 or BRCA2 variants (78 and 21 cases, respectively). BRCA1 variants (5382insC, 300T/G, 4153delA, 2080delA, 3819del5, and 3875del4), highly prevalent in Slavs, accounted for 47.4% of total; full list of the BRCA variants is given in the Supplementary Table 1.

### Repertoire and Distribution of Somatic Variants in HGSOC

FFPE primary tumors from 99 patients with relapse of BRCA-deficient ovarian cancer and paired blood samples were sequenced using CCMSeq panel. The detected somatic variants were absent in paired blood samples. Only somatic variants affecting the amino acid sequence of a protein were considered. A total of 106 somatic variants were identified across six genes (*TP53, APC, BAX, KRAS, CDKN2A, CTNNB1*) in 96/99 patients (see the variants destination and ClinVar classification in the Supplementary Table 1).

**Figure 1:**
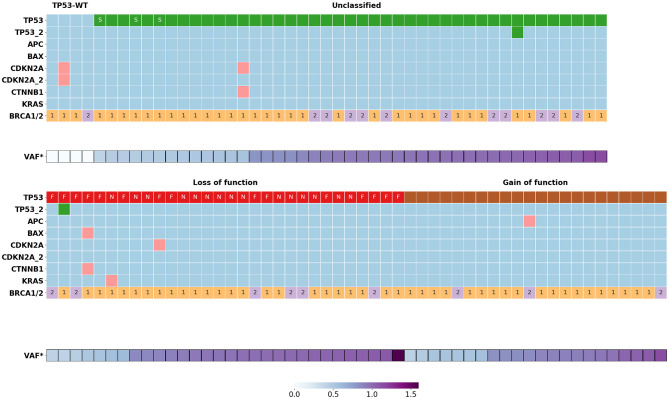
Somatic mutations distribution in *BRCA1/2* associated HGSOC (*n* = 99). VAF*, normalized variant allele frequency of mutant *TP53* allele; S, variants of donor/acceptor splice sites; F, frame shift mutations; N, nonsense mutations; 1, *BRCA1* mutations; 2, *BRCA2* mutations.

*TP53* variants were found in 95 patients with HGSOC. In two cases, the tumor cells carried two *TP53* variants. *TP53* variants were grouped into three categories: GOF (22), LOF (30), and “unclassified” (45) (Figure 1, Table 1). We compared the results with the distribution of GOF, LOF and “unclassified” variants in the HGSOC variant set of the IARC TP53 database. The frequency of LOF variants (261/1249, 21%) in this group was significantly lower (*p* < 0.01).

**Table 1:** Distribution of various types of somatic mutations.

**Gene**	**Type of mutation**	**BRCA1, *n* = 78**	**BRCA2, *n* = 21**	**IARC *TP53* database Unknown BRCA, *n* = 1249**
*TP53*	Missense	47	11	
	frameshift deletions	11	4	
	Nonsense	13	2	
	other	4	3	
*TP53*	GOF^*^	19 (25%)	3 (15%)	271 (22%)
	LOF^*^	24 (32%)^**^	6 (30%)^**^	261 (21%)^**^
	Unclassified	32 (42%)	11 (55%)	717 (57%)
	Wild type	3	1	
APC	nonsense	-	1	
BAX	frameshift	1	-	
KRAS	missense (pathogenic)	1	-	
CDKN2A	**missense**			
	unknown	3	-	
	pathogenic	1	-	
CTNNB1	missense (pathogenic)	2	-	

* *The percentage of mutations of various types is determined only for cases with mutant TP53*.

** *The percentage of LOF mutations in BRCA1/2-deficient tumors is significantly higher than in the reference group not stratified by BRCA status, p < 0.01*.

There were no significant differences between the distribution of various types of *TP53* variants in BRCA1- and BRCA2-deficient HGSOC. Some of the “unclassified” *TP53* variants (16/45) were annotated in the ClinVar database as pathogenic, the functional significance of the remaining variants unknown. Most of the “unclassified” variants (38/45) were amino acid substitutions located in the DNA-binding domain (DBD, 34 variants), C-terminal tetramerization domain (2 variants) or interdomain spacer (residue 93 between Transactivation Domain and DBD, 2 variants). The rest of the “unclassified” variants were those of donor/acceptor splice sites (3), of splice regions (3) and disruptive inframe deletion (1).

The majority of variants (58/97) identified in *BRCA1/2* associated HGSOC were “unclassified” and GOF missense. We used the IARC database information to further characterize all missense variants by the presence of transcriptional and GOF activity in the corresponding mutants. In respect to transcriptional activity, the common missense variants (54/58, 93.1%) were classified as “nonfunctional,” with the rest being “partially functional” (3) and “functional” (1). Mutants with preserved transcriptional activity were from the “unclassified” group. For most *TP53* variants, there were no data on their GOF properties. Petitjean et al. systematized information from the IARC database on GOF properties of 103 TP53 mutants (16). The authors identified three categories of GOF activity: (1) interference with p73, (2) transactivation of genes repressed by wild-type p53, and (3) cooperation with oncogenes for the transformation of mouse embryonic fibroblast cells. In our study, a variant was classified as GOF if the corresponding mutant had at least one of the GOF activities. According to these criteria, 6 of 43 “unclassified” missense variants were determined as GOF.

Six samples had nine concomitant variants in non-*TP53* genes such as *APC* (1), *BAX* (1), *KRAS* (1), *CDKN2A* (4), *CTNNB1* (2) (Figure 1, Supplementary Table 1). There was no significant association between the occurrence of these mutations and the clinical outcome of the corresponding patients, possibly due to the small size of this patient group.

### Loss of Heterozygosity of TP53

For samples carrying somatic variants, we determined the percentage of tumor cells with loss of heterozygosity (LOH). To this end, for each mutation, the variant allele frequency (VAF) was calculated as the ratio of the variant allele reads number to the total number of reads. The tumor content estimated by histological sections assay was used to normalize VAF as :

$$\begin{array}{l} Normalized\;VAF\;=\;\frac{VAF}{\%\;Tumor\;cells} \end{array}$$

The number of variant allele reads is (1+*K*) × *T*, where T is the number of tumor cells in the sample, and k is the proportion of cells with a LOH, so that

$$\begin{array}{l} Normalized\;VAF\;=\;\frac{\left(1+k\right)}{2} \end{array}$$

**Figure 2:**
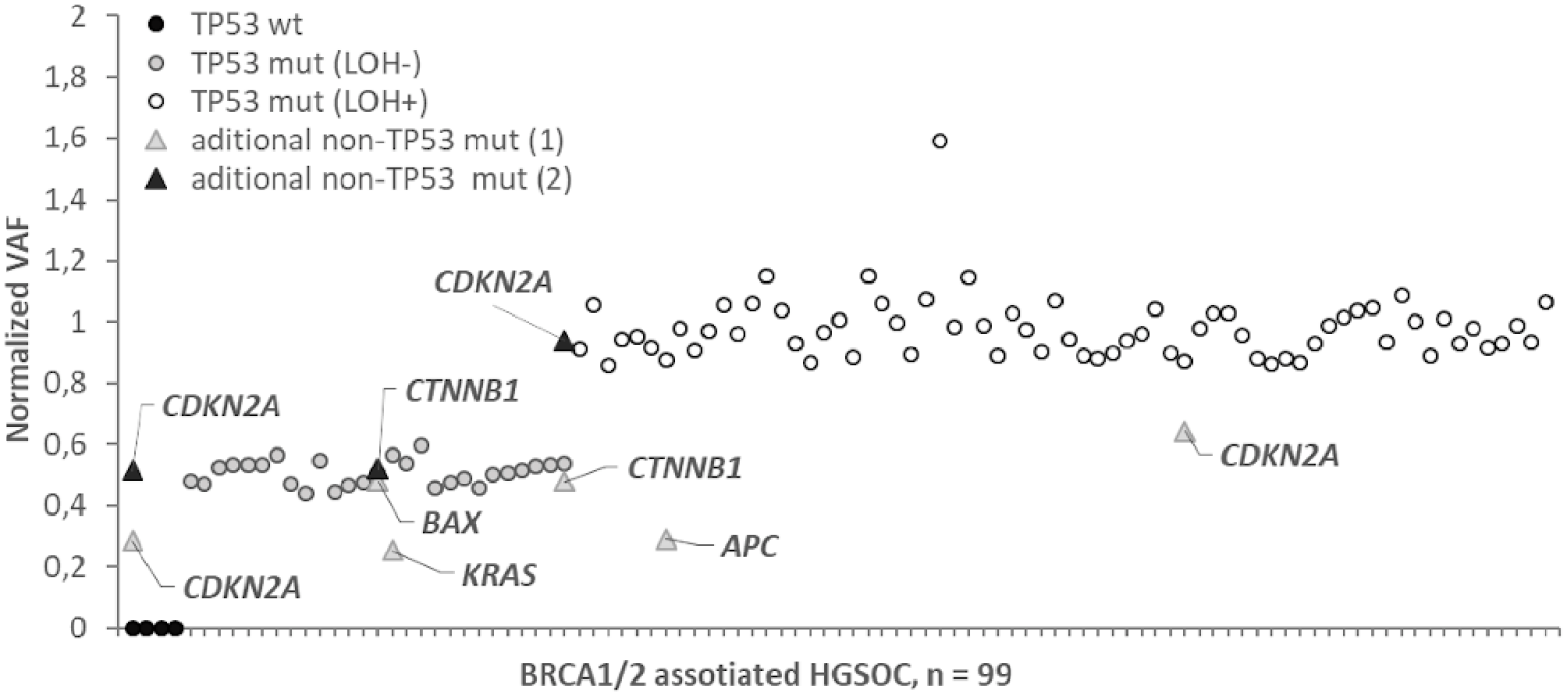
Normalized variant allele frequency (normalized VAF) for somatic mutation in *BRCA1/2* associated HGSOC. Normalized VAFs of 0.5 or 1 correspond to a percentage of LOH positive tumor cells of 0 or 100%. Normalized VAFs of <0.5 indicate that the mutation is contained in the subclones of the tumor; the LOH status for these mutations is not determinable.

For all HGSOC samples with one exception, normalized VAFs were either 0.50 ± 0.05 (29/94) or 1.00 ± 0.08 (65/94), which corresponds to the proportion of tumor cells with LOH, k = 0 or k = 100% (Figure 2). For one sample carrying a single nucleotide deletion in *TP53*, VAF was 1.6, most likely caused by a deviation in the ratio of amplified alleles at the library preparation stage. We found the rate of the tumors with the LOH-positive variant of TP53 to be about 71% (68/95). Most of the nonsense *TP53* variants (14/15, 93%) were LOH-positive. Among other types of variants, the LOH frequency ranged from 60 to 70% (Figure 1).

We did not observe intermediate values 0 < k < 1 for *TP53* variants, while for 3/9 cases in the APC (1), KRAS (1), CDKN2A (1) genes, the normalized VAF was 0.23–0.37, indicating that only subclones of tumor cells carry these somatic variants.

### Immunohistochemical Assay of p53

There is a common agreement that both types of abnormal p53 expression (high and low, or absent) are correlated with mutant p53. The expression level is usually determined by the content of p53 positively stained nuclei. In various studies of HGSOC, the cut-off for high p53 expression levels indicating mutation ranged from 50 to 85% (17–19). In the present study, the cut-off was 70%, as described by Cole et al. (20). We carried out p53 immunohistochemistry for 68/99 patients, with 56 cases (82.3%) found to have abnormal p53 expression (Table 2 and Supplementary Table 1). In addition, we searched for differences in p53 protein levels between patients with GOF, LOF, and “unclassified” variants. Most of the analyzed samples with GOF variants (11/14) were p53 high-positive, while all analyzed tumors carrying LOF variants (9) were low-positive or negative. For the “unclassified” mutants, abnormal p53 expression was found in 31 of 40 cases only. The rest of the “unclassified” tumors had normal (intermediate) p53 expression with the actual number of nuclei staining positive for p53 in the “intermediate” category ranging from 30 to 56% (Supplementary Table 1). For the samples with wild-type *TP53*, only one had normal expression (65% of positive nuclei), while three showed low expression or absence of p53.

**Table 2:** Immunohistochemical classification p53 status for tumor samples with various types of somatic *TP53* mutations.

	**Type of** ***TP53*** **mutation**			
**p53 staining**	**GOF**	**LOF**	**Unclassified**	**Wild Type**
**Negative**	4	8	22	2
**Positive**				
High	11	-	6	-
Intermediate	-	-	9	1
Low	-	1	3	1

## Discussion

The frequency and spectrum of *TP53* variants are highly variable and depend on the type of cancer (2). It is not yet clear what spectrum of *TP53* variants is specific for *BRCA1/2* associated HGSOC. In our study, we first examined the frequency of various types of *TP53* variants in HGSOC patients with a germline *BRCA1* or *BRCA2* pathogenic variants.

Recently, conflicting evidence has emerged on the association of mutated *TP53* type with platinum treatment resistance (21–24). To exclude the possible influence of this factor, only chemo-naive and chemo-sensitive patients with PFI > 6 months were taken in this study.

Our results showed that the frequency of true LOF variants leading to the truncated protein among *BRCA1/BRCA2* carriers with HGSOC was significantly higher than in reference cohort with HGSOC not stratified by BRCA status and chemo-sensitivity (30.3 vs. 21.0%, *p* = 0.01). This finding overlaps with the results of the study by Dumay et al. (25), in which basal-like breast tumors displayed significantly more truncating variants than luminal tumors (43 vs. 25%). Breast cancer with *BRCA1* (but not *BRCA2*) mutations is known to typically have a basal phenotype. Apparently, there is a causal relationship between an increase in the frequency of truncating variants and *BRCA1* alteration.

Truncating variants usually result in loss of any activity of wild-type p53. In contrast, functional outcomes of *TP53* missense variants can be very diverse: LOF, acquisition of oncomorphic function (GOF), or no effect. According to IARC annotation, in our study, the majority (93%) of the missense mutants lacked transactivation activity (26), whereas GOF was described for 28 mutants (22, with conventional GOF variants and 6, with “unclassified” variants) (16). However, it should be kept in mind that most experimental studies of GOF were focused on testing the frequent *TP53* variants clustered at codons 175, 245, 248, 249, 273, and 282. For non-hot spot *TP53* variants, there are insufficient data on their GOF properties, making it impossible to perform a system analysis of GOF for all *TP53* variants.

We used the expression level of the TP53 gene as an indirect marker of mutant p53 with GOF properties, as reviewed by other authors (27, 28). It is conceivable that mutant p53 accumulation in tumors is crucial to exert its GOF in carcinogenesis (29–32). To characterize *TP53* variants with respect to p53 protein expression, we performed IHC with 68 samples. Most of the analyzed samples with GOF variants (11/14) were p53 high-positive, while all analyzed tumors carrying LOF variants (9) were p53 low-positive or negative (Table 2). These results suggest the absence or low level of expression to predict a loss of p53 function, with a sensitivity of 100% and a specificity of 73%. According to this criterion, 25 of 40 IHC tested “unclassified” missense variants with the low and absent level of p53 expression can be defined as probable LOF. Importantly, only 6/40 “unclassified” mutants were p53 high-positive, and the rest (9) had an intermediate level of protein expression. If an elevated expression of mutant p53 is a factor of pro-oncogenic activity, then 9 variants in the samples with a normal level of p53 expression cannot be classified as GOF.

Four BRCA-deficient HGSOC (4/99) did not have somatic variants in *TP53*, but, surprisingly, 3/4 “normal” samples did show abnormal (low or absent) p53 expression levels, i.e., three of four tumors lost p53 function. A possible reason for the loss of p53 function is the deregulation of p53 stability, for example, through the amplification of *MDM2*, the protein of which regulates p53 proteasome degradation (33).

An important feature of p53 function is the integrity of the second *TP53* allele. Most often, inactivation of the second allele occurs through copy-neutral LOH. In our study, LOH was determined based on the normalized VAF. Interestingly, for all LOH-negative and LOH-positive samples, the proportion of tumor cells with LOH was close to 0 and 100%, respectively (with one exception). Based on these findings, we hypothesized that there are no significant sub-clonal populations of cells with different LOH-status of *TP53* in primary *BRCA1/2*-associated HGSOC. This assumption is consistent with the driver role of p53 in carcinogenesis. Thus, potential intratumor heterogeneity and clonal evolution under the pressure of treatment or metastasis will result from the selection of concomitant non-*TP53* somatic variants like *BRCA1/2* and other genes.

Since LOH is a sign of driver variant, it can be assumed that the proportion of LOH-positive variants of *TP53* will be close to 100%. It was the case for tumors with nonsense TP53 variants, where the incidence of LOH was 93% (14/15). In other cases, the rate of LOH-positive variants was about 67%. The relatively low frequency of LOH can be explained by the existence of an alternative way to disable the second *TP53* allele through the interference of p53 missense mutants with wild-type p53 (dominant-negative effect, DNE) (34).

In addition to *TP53*, we sequenced the loci of genes containing frequent somatic variants specific to solid tumors, including ovarian cancer. Additional somatic variants were found in six patients. A total of 9 variants were found in the genes *APC* (1), *BAX* (1), *KRAS* (1), *CDKN2A* (4), *CTNNB1* (2). Three out of the six patients (including one patient with normal p53) had variants in the *CDKN2A* gene whose protein products p14ARF and p16INK4a act as tumor suppressors due to negative regulation of the cell cycle (35).

Variants of *BAX*, coding core regulators of apoptosis and (or) *APC* and *CTNNB1* were detected in three tumors. The products of both genes (*APC* and *CTNNB1*) are components of the Wnt-signaling pathway, an important element in the regulation of embryogenesis and cell differentiation. (36). Studies of cancer genetics showed that genes encoding proteins of the Wnt pathway are frequent targets for mutational alterations in various cancers, including colon, prostate, breast, and ovarian cancer (37). The aberrant activation of the APC/b-catenin pathway is suggested to be restricted to endometrioid ovarian cancer. It is possible that these two samples were incorrectly classified as adenocarcinoma or contain subclones with different histological differentiation.

Variants in other classical oncogenes specific for solid tumors were not detected except for the only specimen carrying the *KRAS* variant. Based on the normalized VAFs of *TP53* and *KRAS* variants (0.51 and 0.23, correspondingly), two equally possible clonal architecture of the tumor can be suggested. First, the tumor contains clonal driver mutation of *TP53* (without LOH) and subclonal (affecting about 50% of cells) mutation *KRAS* likely to have occurred later in tumor evolution. Second, mutations of *TP53* (with LOH) and *KRAS* are independent subclones (each affecting about 50%). Perhaps the tumor is a mix of high and low-grade serous cancer (since the primary KRAS mutations are characteristic of low-grade ovarian carcinoma). Then, the question arises of the multiclonal origin of the tumor. According to the current concept of serous carcinoma pathogenesis, the second option seems less likely. However, it should not be excluded.

According to the latest data, the same variants of *TP53* exert different properties depending on the origin, stage, and molecular profile of a tumor. It was previously shown that patients with HGSOC carrying concurrent somatic variants in *TP53* and additional driver oncogenes had a worsened prognostic signature (reduced PFI, time to recurrence and OS) (18). On the other hand, it is known that the p53 mutants cooperate synergistically with other oncogenes (RAS, TGF-b), causing a more aggressive cancer (38–42). In our study, most samples of HGSOC did not have additional (other than *TP53* and *BRCA1/2)* variants in oncogenes, which seems to be a favorable prognostic factor. Apparently, under such microenvironment, potential GOF (pro-oncogenic) properties are less likely to manifest.

This study has potential limitations. (1) As a reference group, the set of variants specific for HGSOC from the IARC TP53 database was used. For most cases of the reference group, ethnicity and BRCA status were not defined. It is likely that some reference cases of HGSOC are associated with BRCA. A case-control study (BRCA1/2 associated vs. sporadic HGSOC) has more sensitivity to detect differences in the compared samples (would be more preferable). The source of bias is the probable ethnicity heterogeneity of the compared samples and, consequently, the heterogeneous structure of inherited genetic factors, for example, prevalence and spectrum of *BRCA1/2* mutations. Therefore, our findings are supposed to be confirmed by studies with large samples adjusted to ethnicity. (2) To determine non-TP53 somatic mutations, a target panel was used that covers the loci most frequently mutated in solid cancers. However, relatively rare cancer genes have not been sequenced although it can be expected that some of them might have clinical relevance.

## Conclusions

We have focused on molecular profiling of chemo-naive and platinum-sensitive HGSOC with germline *BRCA1/2* variants. Using NGS we have analyzed both the set of *TP53* variants and somatic variants of other genes involved in carcinogenesis. Our findings showed that somatic TP53 variant or inhibition of wild-type p53 expression was observed in almost 100% of cases with *BRCA1/2* associated HGSOC. Rare exceptions are accompanied by variants in other genes of the cell cycle, confirming earlier observations that the negative regulation of cell cycle checkpoints is the main hallmark of BRCA-deficient class of HGSOC. With missense variants predominating among *TP53*, the proportion of truncating variants is significantly higher than with a mixed (in terms of BRCA mutations and sensitivity to platinum) cohort of HGSOC. LOH is typical for *TP53* nonsense variants only, while for other types of variants, there is no pattern in the distribution of LOH. Loss of transcription activities is a common property of missense p53 mutants. There are several indirect signs (normal or low expression of mutants p53, low incidence of concomitant oncogenes mutations) indicating the low manifestation of the GOF properties of *TP53* variants in BRCA1/2 associated HGSOC. Due to the driver role of *TP53*, its variants will persist in all tumorous subclones during treatment or metastasis. This knowledge can be useful in the management of patients with advanced ovarian cancer.

## Data Availability Statement

The datasets presented in this study can be found in online repositories. The names of the repository/repositories and accession number(s) can be found below: https://www.ncbi.nlm.nih.gov/sra/PRJNA612603, Accession no: PRJNA612603.

## Ethics Statement

The studies involving human participants were reviewed and approved by the Institute of Molecular Biology and Biophysics Ethics Committee (Protocol: # 1 dated March 14, 2017). The patients/participants provided their written informed consent to participate in this study.

## Author Contributions

UB and LG: conceptualization. UB: funding acquisition. DL, NK, and AMA: resources (patients and clinical description). EK, AMA, and NK: investigation. AK, AM, and AA: software. UB and AK: formal analysis. MF: supervision. UB and LG: writing—original draft. All authors: writing—review and editing.

## Conflict of Interest

The authors declare that the research was conducted in the absence of any commercial or financial relationships that could be construed as a potential conflict of interest.

## Supplementary Material

Supplementary Table 1 List of *BRCA1/2* germline mutations and somatic mutations in the HGSOC samples.

Supplementary Table 2 List of the custom NGS panel loci.

## References

[1] Kandoth C McLellan MD Vandin F Ye K Niu B Lu C. Mutational landscape and significance across 12 major cancer types. Nature. (2013) 502:333–9. 10.1038/nature12634 24132290 PMC3927368

[2] Leroy B Anderson M Soussi T. TP53 mutations in human cancer: database reassessment and prospects for the next decade. Hum Mutat. (2014) 35:672–88. 10.1002/humu.22552 24665023

[3] Muller PA Vousden KH. Mutant p53 in cancer: new functions and therapeutic opportunities. Cancer Cell. (2014) 25:304–17. 10.1016/j.ccr.2014.01.021 24651012 PMC3970583

[4] Liu G Parant JM Lang G Chau P Chavez-Reyes A El-Naggar AK. Chromosome stability, in the absence of apoptosis, is critical for suppression of tumorigenesis in Trp53 mutant mice. Nat Genet. (2004) 36:63–8. 10.1038/ng1282 14702042

[5] Dittmer D Pati S Zambetti G Chu S Teresky AK Moore M. Gain of function mutations in p53. Nat Genet. (1993) 4:42–6. 10.1038/ng0593-42 8099841

[6] Lang GA Iwakuma T Suh Y Liu G Rao VA Parant JM. Gain of function of a p53 hot spot mutation in a mouse model of Li-Fraumeni syndrome. Cell. (2004) 119:861–72. 10.1016/j.cell.2004.11.006 15607981

[7] Schulz-Heddergott R Stark N1 Edmunds SJ Li J Conradi L Bohnenberger H. Therapeutic ablation of gain-of-function mutant p53 in colorectal cancer inhibits Stat3-mediated tumor growth and invasion. Cancer Cell. (2018) 34:298–314.e7. 10.1016/j.ccell.2018.07.004 30107178 PMC6582949

[8] Zhang Y Xiong S Liu B Pant V Celii F Chauet G. Somatic Trp53 mutations differentially drive breast cancer and evolution of metastases. Nat Commun. (2018) 9:3953. 10.1038/s41467-018-06146-9 30262850 PMC6160420

[9] Bertheau P Lehmann-Che J Varna V Dumay A Poirot B Porcher R. p53 in breast cancer subtypes and new insights into response to chemotherapy. Breast. (2013) 2:S27–9. 10.1016/j.breast.2013.07.005 24074787

[10] Alsop K Fereday S Meldrum C de Fazio A Emmanuel C George J. BRCA mutation frequency and patterns of treatment response in BRCA mutation-positive women with ovarian cancer: a report from the Australian Ovarian Cancer Study Group. J Clin Oncol. (2012) 30:2654–63. 10.1200/JCO.2011.39.8545 22711857 PMC3413277

[11] Prakash R Zhang Y Feng W Jasin M. Homologous recombination and human health: the roles of BRCA1, BRCA2, and associated proteins. Cold Spring Harb Perspect Biol. (2015) 7:a016600. 10.1101/cshperspect.a016600 25833843 PMC4382744

[12] Popova T Manié E Rieunier G Caux-Moncoutier V Tirapo C Dubois T. Ploidy and large-scale genomic instability consistently identify basal-like breast carcinomas with BRCA1/2 inactivation. Cancer Res. (2012) 72:5454–62. 10.1158/0008-5472.CAN-12-1470 22933060

[13] Shi SR Cote RJ Wu L Liu C Datar R Shi Y. DNA extraction from archival formalin-fixed, paraffin-embedded tissue sections based on the antigen retrieval principle: heating under the influence of pH. J Histochem Cytochem. (2002) 50:1005–11. 10.1177/002215540205000802 12133903

[14] Kechin AA Boyarskikh UA Ermolenko NA Tyulyandina AS Lazareva DG Avdalyan AM. Loss of heterozygosity in BRCA1 and BRCA2 genes in patients with ovarian cancer and probability of its use for clinical classification of cariations. Bull Exp Biol Med. (2018) 165:94–100. 10.1007/s10517-018-4107-9 29797126

[15] Brachova P Mueting SR Carlson MJ Goodheart MG Button AM Mott SL. TP53 oncomorphic mutations predict resistance to platinum- and taxane-based standard chemotherapy in patients diagnosed with advanced serous ovarian carcinoma. Int J Oncol. (2015) 46:607–18. 10.3892/ijo.2014.2747 25385265 PMC4277253

[16] Petitjean A Achatz MI Borresen-Dale AL Hainaut P Olivier M. TP53 mutations in human cancers: functional selection and impact on cancer prognosis and outcomes. Oncogene. (2007) 26:2157–65. 10.1038/sj.onc.1210302 17401424

[17] Yemelyanova A Vang R Kshirsagar M Lu D Marks MA Shih IM. Immunohistochemical staining patterns of p53 can serve as a surrogate marker for TP53 mutations in ovarian carcinoma: an immunohistochemical and nucleotide sequencing analysis. Mod Pathol. (2011) 24:1248–53. 10.1038/modpathol.2011.85 21552211

[18] Lassus H Leminen A Lundin J Lehtovirta P Butzow R. Distinct subtypes of serous ovarian carcinoma identified by p53 determination. Gynecol Oncol. (2003) 91:504–12. 10.1016/j.ygyno.2003.08.034 14675668

[19] Köbel M Reuss A du Bois A Kommoss S Kommoss F Gao D. The biological and clinical value of p53 expression in pelvic high-grade serous carcinomas. J Pathol. (2010) 222:191–98. 10.1002/path.2744 20629008

[20] Cole AJ Dwight T Gill AJ Dickson KA Zhu Y Clarkson A. Assessing mutant p53 in primary high-grade serous ovarian cancer using immunohistochemistry and massively parallel sequencing. Sci. Rep. (2016) 6:26191. 10.1038/srep26191 27189670 PMC4870633

[21] Garziera M Roncato R Montico M De Mattia E Gagno S Poletto E. New challenges in tumor mutation heterogeneity in advanced ovarian cancer by a targeted next-generation sequencing (NGS) Approach. Cells. (2019) 8:E584. 10.3390/cells8060584 31197119 PMC6627128

[22] Kang HJ Chun SM Kim KR Sohn I Sung CO. Clinical relevance of gain-of-function mutations of p53 in high-grade serous ovarian carcinoma. PLoS ONE. (2013) 8:e72609. 10.1371/journal.pone.0072609 23967324 PMC3742716

[23] Brachova P Thiel KW Leslie KK. The consequence of oncomorphic*TP53* mutations in ovarian cancer. Int. J. Mol. Sci. (2013) 14:19257–19275. 10.3390/ijms140919257 24065105 PMC3794832

[24] Tuna M Ju Z Yoshihara K Amos CI Tanyi JL Mills GB. Clinical relevance of TP53 hotspot mutations in high-grade serous ovarian cancers. Br J Cancer. (2019) 122:405–12. 10.1038/s41416-019-0654-8 31780779 PMC7000721

[25] Dumay A Feugeas JP Wittmer E Lehmann-Che J Bertheau P Espié M. Distinct tumor protein p53 mutants in breast cancer subgroups. Int J Cancer. (2013) 132:1227–123. 10.1002/ijc.27767 22886769

[26] Kato S Han SY Liu W Otsuka K Shibata H Kanamaru R. Understanding the function-structure and function-mutation relationships of p53 tumor suppressor protein by high-resolution missense mutation analysis. Proc Natl Acad Sci USA. (2003) 100:8424–9. 10.1073/pnas.1431692100 12826609 PMC166245

[27] Muller PA Vousden KH. p53 mutations in cancer. Nat Cell Biol. (2013) 15:2–8. 10.1038/ncb264 23263379

[28] Freed-Pastor WA Prives C. Mutant p53: one name, many proteins. Genes Dev. (2012) 26:1268–86. 10.1101/gad.190678.112 22713868 PMC3387655

[29] Terzian T Suh YA Iwakuma T Post SM Neumann M Lang GA. The inherent instability of mutant p53 is alleviated by Mdm2 or p16INK4a loss. Genes Dev. (2008) 22:1337–44. 10.1101/gad.1662908 18483220 PMC2377188

[30] Walerych D Lisek K Sommaggio R Piazza S Ciani Y Dalla E. Proteasome machinery is instrumental in a common gain-of-function program of the p53 missense mutants in cancer. Nat Cell Biol. (2016) 18:897–909. 10.1038/ncb3380 27347849

[31] Dong P Karaayvaz M Jia N Kaneuchi M Hamada J Watari H. Mutant p53 gain-of-function induces epithelial-mesenchymal transition through modulation of the miR-130b-ZEB1 axis. Oncogene. (2013) 32:3286–95. 10.1038/onc.2012.334 22847613 PMC3705163

[32] Freed-Pastor WA Mizuno H Zhao X Langerød A Moon S Rodriguez-Barruecoet R. Mutant p53 disrupts mammary tissue architecture via the mevalonate pathway. Cell. (2012) 148:244–58. 10.1016/j.cell.2011.12.017 22265415 PMC3511889

[33] Ahmed AA Etemadmoghadam D Temple J Lynch AG Riad M Sharma R. Driver mutations in TP53 are ubiquitous in high grade serous carcinoma of the ovary. J Pathol. (2010) 221:49–56. 10.1002/path.2696 20229506 PMC3262968

[34] Brachmann RK Vidal M Boeke JD. Dominant-negative p53 mutations selected in yeast hit cancer hot spots. Proc Natl Acad Sci USA. (1996) 93:4091–5. 10.1073/pnas.93.9.4091 8633021 PMC39492

[35] Fontana R Ranieri M La Mantia G Vivo M. Dual role of the alternative reading frame ARF protein in cancer. Biomolecules. (2019) 9:E87. 10.3390/biom9030087 30836703 PMC6468759

[36] Nusse R Clevers H. Wnt/β-catenin signaling, disease, and emerging therapeutic modalities. Cell. (2017) 169:985–9. 10.1016/j.cell.2017.05.016 28575679

[37] Zhan T Rindtorff N Boutros M. Wnt signaling in cancer. Oncogene. (2017) 36:1461–73. 10.1038/onc.2016.304 27617575 PMC5357762

[38] Oren M Rotter V. Mutant p53 gain-of-function in cancer. Cold Spring Harb Perspect Biol. (2010) 2:a001107. 10.1101/cshperspect.a001107 20182618 PMC2828285

[39] Adorno M Cordenonsi M Montagner M. A Mutant-p53/Smad complex opposes p63 to empower TGFβ-induced metastasis. Cell. (2009) 137:87–98. 10.1016/j.cell.2009.01.039 19345189

[40] Jia S Zhao L Tang W Luo Y. The gain of function of p53 mutant p53S in promoting tumorigenesis by cross-talking with H-RasV12. Int J Biol Sci. (2012) 8:596–605. 10.7150/ijbs.4176 22553460 PMC3341601

[41] Zhang X Qi Z Yin H Yang G. Interaction between p53 and Ras signaling controls cisplatin resistance via HDAC4- and HIF-1α-mediated regulation of apoptosis and autophagy. Theranostics. (2019) 9:1096–114. 10.7150/thno.29673 30867818 PMC6401400

[42] Liang F Ren C Wang J Wang S Yang L Han X. The crosstalk between STAT3 and p53/RAS signaling controls cancer cell metastasis and cisplatin resistance via the Slug/MAPK/PI3K/AKT-mediated regulation of EMT and autophagy. Oncogenesis. (2019) 8:59. 10.1038/s41389-019-0165-8 31597912 PMC6785561

